# The experiences of family members witnessing the diminishing drinking of a dying relative in hospital: A narrative inquiry

**DOI:** 10.1177/02692163231164452

**Published:** 2023-03-27

**Authors:** Annie Pettifer, Sean Hughes

**Affiliations:** 1 University of Birmingham, Birmingham, UK; 2 Lancaster University, Lancashire, UK

**Keywords:** Pragmatism, narrative medicine, fluid therapy, drinking, palliative care, terminal care, family, caregiver

## Abstract

**Background:**

The optimal management of diminishing drinking at the end of life is contentious. Clinicians and family members may understand the phenomenon differently and hold divergent priorities regarding care. Family members can be distressed by diminishing drinking and its management, particularly when in a hospital environment.

**Aim:**

To explore the experiences of family members when witnessing the diminishing drinking of a dying relative.

**Design:**

A narrative inquiry methodology, derived from pragmatism.

**Setting and participants:**

Thirteen recently bereaved family members were recruited through the bereavement services of three UK hospitals. Inclusion criteria included having an adult relative who died in hospital of any diagnosis more than 48 hours from admission and who had had noticeable diminishing drinking.

**Findings:**

Participants experienced diminishing drinking as an unfolding process that was part of overall decline. They all believed it to be detrimental. Three groups of responses were identified: promoting, accepting and ameliorating. Supportive measures included offering equipment to support drinking, staff being present and communicating about expectations and care management aims.

**Conclusions:**

There is potential to improve family members’ experiences through re-conceptualisation of diminishing drinking aligned to their experiences, supporting family members by listening to their experiences with insight and strengthening their agency within the management of their relatives with diminishing drinking.

**What is already known about the topic?**

- The diminishing drinking of dying people and its clinical management often concerns the families of dying patients, particularly when in hospital.
- Issues around hydration at the end of life are a research priority for family members, clinicians and people approaching the end of their lives.

**What this paper adds?**

- Family members experience diminishing drinking as the culmination of a process starting much earlier in the disease trajectory.
- Family members view dehydration as detrimental to dying people, including at the very end of life.
- Family responses to diminishing drinking vary with their beliefs, values and expectations of care.

**Implications for practice, theory or policy**

- Family members value communication with healthcare professionals about the diminishing drinking of their family members.
- The experience of family members of dying patients with diminishing drinking in hospital could be improved by measures to increase their agency to support their relatives, for example through access to kitchens.
- Diminishing drinking can be conceptualised alongside diminishing intake as part of overall progressive decline.

## Introduction

As people with advanced, life-limiting conditions become progressively less conscious and enter the last few days of their lives, their everyday drinking of liquids normally diminishes and sometimes ceases altogether.^1^ Family members and close friends will inevitably notice and may be troubled by this. Since palliative care includes the promotion of the quality of life of such families,^2^ the exploration of their concerns lies within the scope of palliative care endeavour. Issues relating to hydration have been identified as a research priority by dying patients, family members and healthcare professionals within the UK.^3,4^ A national survey of bereaved families in the UK suggests that this is particularly the case in hospitals since family members’ satisfaction with the support given to dying relatives to drink is weakest in this setting.^5^

No research specifically exploring the experience of family members who witness diminishing drinking of dying relatives could be identified on review,^6^ although it has been explored as a theme within free text survey data about research priorities in palliative care.^3^ A small body of research has described family members’ experience of diminishing oral intake (encompassing both food and drink) at the end of life. It demonstrates that diminishing oral intake is a source of concern, and sometimes distress to family members.^3,4,7–11^ Furthermore, the views of family members regarding the optimal management of this decline may conflict with those of clinicans.^10,11^ There is literature exploring the impact of the declining eating of people with advanced cancer on family members and relationships.^12^ In addition, some literature has described professionals’ understanding of family members’ concerns about the diminishing oral intake of dying relatives; finding it can be challenging for professionals.^13^ Broader literature has considered professionals’ communication with patients and family members about declining oral intake^14^ and clinically assisted hydration.^15–18^ The latter provides some evidence that attitudes towards clinically assisted hydration are rooted in cultural and religious beliefs.^16–18^

An espoused aim of palliative care is to improve quality of life for dying people and those close to them.^2^ The usefulness of the existing literature for understanding family members’ experience of the diminishing drinking of dying relatives is limited because it focuses on those with a cancer diagnosis^7,19–21^; is commonly undertaken in specialist palliative care^7,19–25^; is often aggregated with diminishing eating^7,8,19–21^; and has been undertaken within countries with developed healthcare systems, limiting generalisability to other contexts. This study aimed to address gaps in existing knowledge by exploring family members’ direct experiences of diminishing drinking of a relative dying in hospital, thereby adding important understanding to a complex area of care.

## Methods

### Research question

The study explored the following questions, framed as a ‘research puzzle’.^20–22^

What are family members’ experiences of their dying relatives’ diminishing drinking in hospital? What sense do they make of the diminishing drinking and how do they react to it? What are their experiences of the healthcare given to their relative and support offered to themselves?

For the purposes of the research, the term ‘family member’ denotes those who are significant to dying people at the end of their lives, regardless of their biological or legal relationship. The term ‘relative’ denotes the person dying, not the family member.

### Study design

The study was designed using the narrative inquiry methodology devised by Clandinin and Connelly,^26–28^ which explores an experience in order to generate new understanding to enhance future experience. The methodology draws on pragmatism, particularly the ontology and epistemology of experience espoused by James,^29^ and developed by Dewey.^30–32^ Clandinin’s narrative inquiry methodology is particularly suited to this research because, like the research puzzle, it is centred on experience. Phenomenology was considered but rejected in favour of narrative inquiry because of the latter’s emphasis on instrumentalism or potential to be a tool for positive change.

### Setting

Recruitment took place within the bereavement services of three acute hospitals in an urban area of the United Kingdom.

### Population

The target population was bereaved family members who had noticed that their relative’s drinking diminished prior to death. The inclusion and exclusion criteria are summarised in Table 1 below.

**Table 1.** Inclusion and exclusion criteria.

Inclusion criteria	Exclusion criteria
People who noticed a dying relative drinking less and less as they approached the end of their life in hospital.	Those whose deceased relative died suddenly and unexpectedly.
People over 18 years old.	Those whose deceased relative was in hospital less than 48 h before their death.
People who were willing and able to be interviewed, normally within 50 mi of the research base.	Those whose relative died under 18 years of age.
People who were able to speak English.	

### Sample

Methodological and epistemological imperatives guided the sample size.^33^ Given Clandinin’s position that ‘experience’ is unique,^28^ a sample cannot be representative of a wider population. Instead, sample size is determined by the purpose of the study. The sample size of published studies that have used Clandinin’s approach varies from 62^34^ to 4.^35^ For this study, a purposive sample of 13 facilitated exploration of the research puzzle.

### Recruitment

A research invitation pack comprising a letter, participant information sheet, expression of interest form and a pre-stamped addressed envelope was added to information routinely given to family members visiting the bereavement administrators within the participating hospitals. Normal practice at the time was for family members of all deceased patients throughout these hospitals to visit the bereavement administrators approximately 3–10 days after the death to collect the death certificate, unless the death was referred to the coroner for investigation in which case the visit was delayed or omitted. To the best of the researchers’ knowledge, each family visiting the bereavement administrators received an invitation to self-select into the study.

Interested family members returned the ‘expression of interest’ form. The researcher contacted them to ascertain if they met the inclusion criteria. 13 of the 14 respondents met the criteria and were invited for interview in a mutually convenient private location. Written informed consent to participate in the study was gained prior to the interview.

### Data collection

Data were collected using narrative interviewing^36^ between December 2018 and July 2019. Participants were invited to tell their story of witnessing the diminishing drinking of their dying relative, starting at the point that they first noticed the decline. An interview guide, piloted with a person who had witnessed the diminishing drinking of a dying relative some years ago provided a reference point. It included the following: the family background and the circumstances of the death, an account of the dying relative’s diminishing drinking, beliefs about diminishing drinking and experiences of healthcare for the dying person and support of the family. One of the authors (AP) conducted all the interviews individually. She is a registered nurse with a clinical background in palliative care, researching as part of a PhD. All except one took place at the participant’s home. Eleven interviews took between an hour and an hour and a half, and two were under an hour. The interviews took place between 3 and 24 weeks after the relative’s death.

### Data analysis

The interview recordings were transcribed verbatim, anonymised and uploaded to Atlas.ti qualitative data analysis software. The researcher analysed the stories of diminishing drinking within the interview transcripts in the light of the research puzzle and of notions of temporality, sociality and spatiality. These parameters are: time and sequence, personal and social context, and the environment within which the narrative unfolds.^26^ Significant points were identified where participants specifically identified them as important or where participants emphasised them using language, tone, emotion or repetition. Excerpts containing significant points were presented as a continuous story or ‘narrative account’.^28^ These significant points were also sorted into the three ‘a priori’ strands within the scope of the research puzzle and then used to identify the areas of resonance within each strand. This process was not the same as identifying commonalities and clustering them as themes as per narrative thematic analysis,^37^ rather it was a process of exploring relations between the experiences of participants. This paper reports on the areas of resonance within each strand.

## Findings

The 13 participants comprised 4 men and 9 women and were family members of 12 deceased people. Table 2 shows key characteristics of participants and key features they offered about their deceased relatives.

**Table 2.** Key characteristics of the deceased relatives as reported by participants.

Characteristics	Variables	*n*
Relationship to deceased	Offspring	8
	Offspring-in-law	1
	Spouse	3
	Friend	1
Age of deceased	90–100	6
	80–90	3
	70–79	1
	60–69	2
Primary diagnosis of deceased	Cancer	3
	Heart disease	1
	Dementia	3
	Respiratory disease	2
	Neurological disease	1
	Infection	1
	Musculo-skeletal disease	1
Cause of admission of deceased	General deterioration	4
	Infection	3
	Constipation	1
	Breathing difficulties	1
	Fall	2
	Collapse	1
Duration of admission of deceased	<1 week	1
	>I week	3
	>2 weeks	6
	>3 weeks	2
Received specialist palliative care during hospital admission	Yes	1
	No	11

The following areas of resonance were found within the data. All names are pseudonyms.

### Experiences of diminishing drinking

Every participant described how their dying relative’s drinking diminished gradually, within a context of general deterioration, culminating in death. The length of this process varied but for most, it occurred over months and years (Mark, Jane, Colin, Irene, Ajinder, Frank, Martina and Estelle). Participants linked the process to other problems associated with the deceased’s diagnosis, such as forgetfulness caused by dementia (Colin) and narrowing of the oesophagus caused by advancing lung cancer (Bernard). The decline was usually punctuated with memorable events or markers of decline, in which treasured habits changed. These included ceasing drinking wine before bed (Martina) and drinking a preponderance of tea (Mark).

The chronology of Colin’s experience was particularly illustrative of the process of diminishing drinking.

It’s quite disturbing to observe. By the time he died, he probably hadn’t had a, a proper drink for a month. I don’t think anyone could have done anything more than they did, and it was part of the decline that had started way back with spitting out bits of his food. (Colin)

For all participants, drinking and drinks were strongly associated with either quality or quantity of life, or both. The following positive impacts were cited: increased strength (Jane, Martina, Susan); increased alertness (Frank, Brenda); increased ability to recover (Jane, Susan); improved skin integrity (Martina, Brenda); and increased likelihood of discharge home (Jane, Susan). The preparing and giving of drinks was seen as a caring; loving associated with giving and receiving comfort. Martina, Susan, Ajinder and Jane specifically visited their relatives at mealtimes.

Conversely, not drinking and consequent dehydration was considered detrimental to health. It was uncomfortable, painful (Frank, Colin) and linked to deterioration and to death (Mark): either as a cause or a herald (Colin, Susan). Almost all participants worried that relatives would be thirsty, and have dry or sore mouths. Some evidenced this with observation (Mark, Rhoda, Irene, Martina), others inferred it (Ajinder, Jane, Colin, Susan).

I knew that the oxygen that she was on was making her very dry. So gave her a sip of water, and it made her cough. So I put the water up to her mouth, and she. . . obviously took a bit of it, I could see her physical reaction was, she wanted, she needed the water, and you could see her tongue come out. And then she just coughed, and obviously it must have caught her, she must be dehydrated, so, I know, I know that she’s very near the end. (Mark)

### Responses to diminishing drinking

All of the participants monitored their relative’s diminishing drinking both before and during their final hospital admission. For some, this was by noticing changes to long-established habits, but also by reading clinical records and the availability of drinks within the hospital environment. As drinking decreased, monitoring became intentional observation.

If I was there during the daytime, she was hardly drinking when I was there, and I’m thinking to myself, “Well I wonder how much she is drinking and if she’s not drinking, how often is the water getting changed?” Visually, there didn’t seem to me, to be much evidence, one, that she was drinking very much, but, two, even that the drinking water was being replenished that often to be fair. (Brenda)

**Figure 1.**
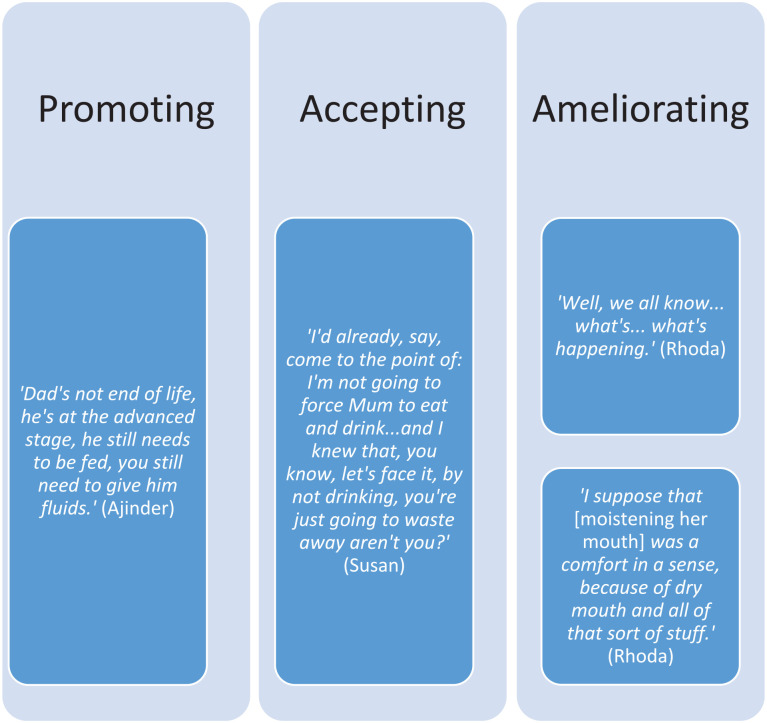
The approaches to diminishing drinking.

Each participant responded to the diminishing drinking with a unique, changeable approach based on their reasoning about what would be best for their relative. These approaches can be categorised as proactively promoting drinking, accepting the effects of diminishing drinking and ameliorating the effects of diminishing drinking (Figure 1). Each approach is considered in turn.

#### Proactively promoting

Ajinder’s response to her father’s diminishing drinking was proactive, ensuring her father drank as long as he was able and henceforth received fluid through assisted means. She adopted this promoting approach early in her father’s declining drinking and continued it resolutely until his death. Ajinder saw herself and her family as his advocates since they were best placed to know his needs. Her role was to ensure he did not die prematurely which, to her, meant before exhausting all treatment options.

Dad was a devout Sikh, and Dad did not want to go before his time, because we believe that the soul remains on earth. Until your time is up, and then you move on to wherever you’ve got to go. . ., and so Dad wanted us to fight for him, and to make sure that Dad did not go before his time, . . . “Whatever there is available medically possible. . .” To get that done for Dad. (Ajinder)

#### Accepting

While most participants encouraged their relatives to drink at times, with the exception of Ajinder, they moved towards acceptance of diminishing drinking as their relative became less well. Susan felt she should respect the choices her mother was making.

At her age, she can do what she likes. It’s not up to me to force feed her or, you know, force drink down her or whatever, um, if she doesn’t want it, she doesn’t want it. At her age, she’s had, she’s had a good innings. (Susan)

Several participants surmised that relatives intentionally reduced their drinking, exerting control by choosing to hasten death. Martina explained that her mother may have wanted to die because she had lost her former glamorous persona.

She used to be a very glamorous woman, very, very, very elegant and beautiful, and um, she hated herself, because of what she looked like, she hated herself because she couldn’t do anything, . . . so whether, in a sense, um, she wasn’t desperate for drink, you know, . . . and she wasn’t desperately hungry either, and she probably, in one way she wanted to go. (Martina)

#### Ameliorating

Some participants moved further than acceptance: to ameliorating the effects of diminishing drinking. Mark did this to ameliorate thirst. Derek invested in beakers, spoons and finally a toothbrush to support his wife to keep her mouth moist. Rhoda, a former nursing assistant, undertook ‘mouth care’ using tools given to her by the nursing staff. Rhoda found the activity personally comforting to undertake, as well as benefitting her mother.

Well, I suppose that was a comfort in a sense, because of dry mouth, and all of that sort of stuff, but yes, so that, for me, that [offering mouth care] was more of a comfort . . . because I was thinking, “At least I can do something”. (Rhoda)

At the time they took an ameliorating approach, Rhoda, Derek, Bernard and Mark unequivocally understood that their relatives were irreversibly dying. Irene also had this understanding and regretted healthcare staff had not had a more ameliorating approach.

He was dying. Why put him through all that agony, moving him off the ward, going to the X-ray, back again, having the tube down? Why didn’t they just take him off the antibiotics completely, and let him, and then if he wanted to drink, give him a drink. (Irene)

**Figure 2.**
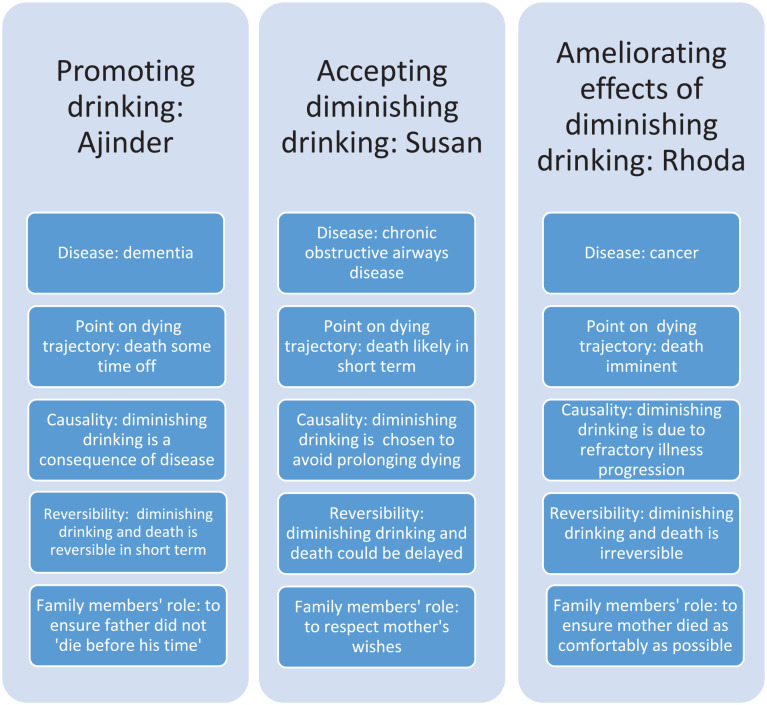
Approach to diminishing drinking of three participants mapped to relatives’ disease, participants’ awareness of their relatives’ dying trajectory, participants’ beliefs about causation and reversibility, and their expectation of their role.

Figure 2 maps the different approaches of three participants to their awareness of their relative’s disease and dying trajectory, their beliefs about causation and reversibility, and their role as they described it. This mapping demonstrates the reasoning underpinning their approach to diminishing drinking, and the significance of their beliefs and understanding about their relative’s illness. For example, Rhoda sought to ameliorate because she and her family perceived diminishing drinking as an inevitable part of irreversible illness progression, while Ajinder maintained that her father was not dying, and that fluid was important to support his recovery.

## Experiences of healthcare and support

Most participants valued healthcare staff monitoring their relative’s drinking and worried if this was not evident. Bernard, Frank, Derek, Brenda and Rhoda particularly appreciated staff assisting drinking through offering water on a teaspoon, in a beaker, syringe or through a straw or providing them with the equipment to do so. Irene appreciated the effort of staff to moisten her husband’s mouth. However, Jane and Marina described times when ward environments were busy and nurses did not support patients to drink, apparently as they had greater priorities.

Mark, Frank and Jane found that the actions of some groups of healthcare staff could be inconsistent with other groups. Jane described this as ‘a disjunct’, Frank as a ‘disconnect’ between nursing and catering staff. Mark gave the following example:

So a nurse had come around, and said, “Oh, is. . ., is. . ., is your mum going to have anything to eat?” and I’m, I’m like, “I think she’s about to die”, so like whether she should have chicken chasseur,[or] what not. . . And I thought that was quite insensitive, but it was almost like the auxiliary nurses. . . [and] the catering staff, there was a disconnect between them and the clinical information. (Mark)

Colin, Irene, Ajinder, Derek and Frank all told stories about the risk of their relative aspirating and its management. They understood the clinical reasons for restricting and adapting drinking, but such changes removed the pleasure associated with drinking and led to tension with healthcare staff over what was best for their dying relatives. Frank illustrated this pleasure when he circumvented the advice:

The day she passed away, she had half a cup of tea on her own, literally picking it up, the nurse didn’t notice, but we gave it to her without the thickener, she had two spoonfuls of sugar in it, and she only ever had one [chuckles] and she said, “This is really nice, I like this.” (Frank)

The relatives of all the participants, except Mark’s, had clinically assisted hydration during their hospital admission, usually described as ‘drips’. Most participants understood the primary purpose of the drip to be rehydration, but some understood it to be giving protein, minerals, nutrition or antibiotics too. Drips were seen as improving health and extending life: ‘I think if she wasn’t on the drips, she’d have gone before Christmas’ (Frank). Despite the advantages, participants also raised negative effects of intravenous fluids; for example, Martina and Rhoda reported that they had caused considerable bruising.

Participants stated that the following measures did, or would have supported them: offering equipment which they could use to support their relative’s drinking (Derek); being present (Frank); and communicating about expectations of their relatives’ dying trajectory and how drinking could best be managed (Susan, Colin, Mark). Mark explained how lack of prognosis clarity left him unsure about drinking:

They told us to come in, because she’s passing away. . . and what we didn’t know, was if we’re looking at days, hours, weeks. So, in the midst of that was, if she’s not having anything to drink now, is it because she only has got two hours left, so it wouldn’t matter?

Frank recalled helpful conversations about decision making around intravenous fluids and end-of-life care in general with doctors and nurses.

The doctor at first said he didn’t want to put her on the drips just to prolong her life basically, which was a hard conversation, but looking back now, . . . that needed to be said. Um, if that had been said a fortnight or so before, it might have been easier. (Frank)

Colin, Susan, Brenda and Jane reported that clinicians had not discussed diminishing drinking with them. While participants wanted clinical information about diminishing drinking and its management, they also drew on their own experience, gained through long relationships, to consider what was best for their relative. Irene illustrates the value of such knowledge: ‘He’d always coughed and choked anyway, so I have always dealt with that’. The risk of her husband choking on a drink did not alarm Irene because it was longstanding.

## Discussion

This study adds insight into how family members perceive diminishing drinking, their response to it and the support they receive from healthcare professionals. The findings echo those in the wider literature about the role of eating in connecting spousal relationships and the consequences of disrupting these during cancer.^12,19^ This study is the first exploration of family members’ experiences of diminishing drinking in isolation.

Participants experienced diminishing drinking as an unfolding process; part of the decline associated with advancing illness. This study found that drinking and drinks have particular significance within families. They all believed diminished drinking to be detrimental, which is consistent with existing research about family members’ perspectives and views on assisted hydration at the end of life.^18,22,38,39^ However, it runs counter to the dominant professional view that diminishing drinking is part of the natural process of dying^14^ and the view that dehydration might positively enhance the comfort of those near to the end of life.^40,41^ Three groups of responses were evident: promoting, accepting and ameliorating the effects. Participants reported positive experiences of healthcare when staff actively supported their relatives to drink, but they also found that other priorities overrode attending to drinking. Tension occurred within families, and between healthcare staff and families over the management of aspiration risk and clinically assisted hydration. Family members valued communication about the diminishing drinking of their dying relative.

Family members are likely to benefit from a re-conceptualisation of diminishing drinking as a phenomenon occurring alongside diminishing eating and progresses with advancing illness and culminates in the last few days of life. This conceptualisation is more consistent with the experience of family members than diminishing drinking being an aspect of the dying process. It is particularly relevant in unpredictable illness trajectories, and where family members have been caring over a long period of decline; all factors that abound in hospitals.^42^ Such a re-conceptualisation may serve to elevate the agency of family members as historians of the course of their relative’s diminishing drinking, illness trajectory and identity prior to hospital admission.

### Considerations for practice

To support family members, clinicians should recognise the significance that drinking has both to individuals and within families. Shared decision making and flexibility regarding the management of diminishing drinking are needed to respect the preferences and deep-seated beliefs of the family. Structured assessment tools and generic care plans are unlikely to be helpful in assessment or managing such complex, unique and temporal experiences because their ability to respond to individuals is limited.^43^ Instead, individual support offered by skilled healthcare clinicians who listen and respond to family members as individuals is more likely to be genuinely supportive.

Clinicians may be able to influence the responses of family members to diminishing drinking through tailored information giving and dialogue about diminishing drinking, its consequences and its clinical management in the light of the disease trajectory and prognosis. There is evidence supporting the value of such information in the preparation and support of family members during withdrawal of life sustaining treatment in intensive care,^44^ and participants in this study valued such discussions. However, seeking to ‘educate’ family members is not appropriate if it seeks to privilege clinical over familial knowledge gained through long relationships. Irene illustrates the value of such knowledge: ‘He’d always coughed and choked anyway. . . so I have always dealt with that’. The risk of her husband choking on a drink did not alarm Irene because it was familiar.

### Considerations for policy

The findings of this study support the promotion of individually tailored care and shared decision making with families within healthcare policy. Hospital policies should actively facilitate family members to care for their relatives experiencing diminishing drinking where possible. This might be achieved through closer working between nurses and catering services, promoting giving family access to kitchens and equipment to care for dying relatives.

Guidance that considers drinking (as well as eating) difficulties more broadly than in just the last few days of life is indicated. Such guidelines may be particularly helpful for hospital care in cases where there is limited predictability of dying.^42^

### Considerations for further research

Family members’ conceptualisation of diminishing drinking is congruent with global research endeavour in which drinking has been considered alongside eating and often earlier than in the last few days of life.^7,8,19–21^ Research into nutritional care at the end of life is likely to be pertinent to diminishing drinking, even if it is not disaggregated from eating.

Future research is needed that develops, implements and evaluates intervention using the insights of this and related studies in order to better support family members of people in hospital with diminishing drinking at the end of their lives. An action research study that co-generates a supportive intervention with family members and healthcare staff would be fruitful in addressing this problem.

### Strengths and limitations

The recruitment method facilitated wide access to family members bereaved through a range of illnesses and medical situations without clinical gatekeeper bias; however, it had limitations. The potential for self-selection sampling bias, particularly from non-response, is acknowledged.^45^ The sampling frame excluded family members who did not visit the bereavement office and no proactive measures to recruit from under-represented groups were taken due to resource constraints. The sample is biased towards female family members (69%) who were all English speaking and whose relatives who died aged over 90 (almost 50%). As such, the findings are not generalisable. However, insight from the experiences of this sample may be transferable.

The scope of this study was intentionally broad so that participants could focus on the issues important to them. This contrasts with much of the literature, which has focused on the family members’ views of the intervention of clinically assisted hydration at the end of life in order to inform clinical decision making.^6^

## Conclusion

Drinking is an ordinary, everyday activity within the lives of individuals and families that, hitherto, has received limited attention as a discrete phenomenon in the palliative care literature. However, the subject is profoundly important to many family members. When drinking wanes, its diminishment brings loss, and the responses of family members to it reflect the unique beliefs, understanding of illness and context of their lives. While healthcare professionals do much to support family members experiencing diminishing drinking of their dying relatives, there are challenges inherent in the hospital environment and healthcare practice that limit the support given. Authentic support requires renewed emphasis on attentive listening to the experience of family members, with insight of its antecedents; respecting their role in preserving and promoting the identity of their relatives in the face of advancing illness; and fostering the agency of family members in the care of those dying with diminishing drinking in hospital environments.
